# Plasma Concentration of Parasite DNA as a Measure of Disease Severity in Falciparum Malaria

**DOI:** 10.1093/infdis/jiu590

**Published:** 2014-10-24

**Authors:** Mallika Imwong, Charles J. Woodrow, Ilse C. E. Hendriksen, Jacobien Veenemans, Hans Verhoef, M. Abul Faiz, Sanjib Mohanty, Saroj Mishra, George Mtove, Samwel Gesase, Amir Seni, Kajal D. Chhaganlal, Nicholas P. J. Day, Arjen M. Dondorp, Nicholas J. White

**Affiliations:** 1 Department of Molecular Tropical Medicine and Genetics; 2 Mahidol-Oxford Tropical Medicine Research Unit, Faculty of Tropical Medicine, Mahidol University, Bangkok, Thailand; 3 Centre for Tropical Medicine, Churchill Hospital, University of Oxford; 4 MRC International Nutrition Group, London School of Hygiene and Tropical Medicine, United Kingdom; 5 Cell Biology and Immunology Group, Wageningen University; 6 Laboratory for Microbiology and Infection Control, Amphia Hospital, Breda, the Netherlands; 7 MRC Keneba, the Gambia; 8 Dev Care Foundation, Dhaka, Bangladesh; 9 Department of Medicine, Ispat Hospital, Rourkela, India; 10 Amani Centre, National Institute for Medical Research, Muheza; 11 Korogwe Research Laboratory, National Institute for Medical Research, Tanga, Tanzania; 12 Hospital Central da Beira; 13 Faculty of Health Sciences, Catholic University of Mozambique, Beira, Mozambique

**Keywords:** *Plasmodium falciparum*, malaria, severe disease, plasma DNA, diagnostic accuracy

## Abstract

In malaria-endemic areas, *Plasmodium falciparum* parasitemia is common in apparently healthy children and severe malaria is commonly misdiagnosed in patients with incidental parasitemia. We assessed whether the plasma *Plasmodium falciparum* DNA concentration is a useful datum for distinguishing uncomplicated from severe malaria in African children and Asian adults. *P. falciparum* DNA concentrations were measured by real-time polymerase chain reaction (PCR) in 224 African children (111 with uncomplicated malaria and 113 with severe malaria) and 211 Asian adults (100 with uncomplicated malaria and 111 with severe malaria) presenting with acute falciparum malaria. The diagnostic accuracy of plasma *P. falciparum* DNA concentrations in identifying severe malaria was 0.834 for children and 0.788 for adults, similar to that of plasma *P. falciparum* HRP2 levels and substantially superior to that of parasite densities (*P* < .0001). The diagnostic accuracy of plasma *P. falciparum* DNA concentrations plus plasma *P. falciparum* HRP2 concentrations was significantly greater than that of plasma *P. falciparum* HRP2 concentrations alone (0.904 for children [*P* = .004] and 0.847 for adults [*P* = .003]). Quantitative real-time PCR measurement of parasite DNA in plasma is a useful method for diagnosing severe falciparum malaria on fresh or archived plasma samples.

Severe falciparum malaria is a major cause of childhood death in sub-Saharan Africa, where it is a common diagnosis in severely ill children. In areas of moderate and high transmission, malaria parasitemia is also common in apparently healthy children. Because of this, the specificity of a blood slide for malaria is poor, and malaria is consequently overdiagnosed. Postmortem studies indicate that nearly 25% of children dying with a clinical diagnosis of cerebral malaria actually died from other causes [1], resulting in the underdiagnosis of other treatable severe infections. Improving the specificity of the diagnosis of severe malaria is therefore a priority. Recent studies showed that careful ophthalmoscopy to detect the retinopathy specific to cerebral malaria [2] and quantitation of plasma concentrations of *Plasmodium falciparum* HRP2 as a measure of parasite biomass [3] separately improved diagnostic accuracy substantially in severe falciparum malaria.

Because *P. falciparum* HRP2 is released at the time of schizont rupture, we investigated the hypothesis that plasma concentrations of DNA (reflecting liberated merozoites and degraded parasites) might also reflect the cumulative parasite burden and, thus, the severity of disease in falciparum malaria. Plasma concentrations of bacterial DNA have been shown to correlate with severity in a number of invasive bacterial infections [4–6]. Several studies have documented the presence of *P. falciparum* DNA in plasma or serum [7–9] but have not examined the relationship between plasma *P. falciparum* DNA concentrations and malaria severity. We reasoned that DNA released into the plasma by schizont rupture or degradation of sequestered parasites might be a better measure of the previously sequestered biomass and, thus, disease severity than peripheral parasitemia. We assessed the diagnostic performance of plasma *P. falciparum* DNA concentration, alone and then combined with plasma *P. falciparum* HRP2 concentration and parasitemia, to distinguish between uncomplicated and strictly defined severe malaria in African children and Asian adults.

## METHODS

### Cases

The pediatric study involved children with symptomatic *P. falciparum* malaria in East Africa. Samples from uncomplicated cases were obtained during longitudinal community studies undertaken between February and August 2008 in Handeni district, northeast Tanzania. These were febrile children with a positive result of a rapid diagnostic test (RDT) for *P. falciparum* lactate dehydrogenase (pLDH) but no clinical signs or laboratory indicators of severe malaria [10]. Samples from severe cases came from children with a positive result of a pLDH RDT and signs of severe malaria consecutively recruited in the Teule (northeast Tanzania) and Beira (central Mozambique) centers of a large multinational randomized controlled trial (AQUAMAT) that compared quinine and artesunate for the treatment of severe malaria [11]. Plasma DNA concentrations were determined for sets of consecutive samples obtained in Handeni during 2008, in Teule during 2007, and Beira during 2009. The adult study involved samples from all nonpregnant adults (≥16 years old) who were admitted to the hospital with slide- or RDT-confirmed falciparum malaria and enrolled in prospective studies at Chittagong Medical College Hospital, Bangladesh (2009–2011), and Ispat General Hospital, Rourkela, India (2011). Uncomplicated and severe cases were defined using modified World Health Organization criteria [12] and all were treated with artesunate either parenterally [11] or orally as part of artemisinin-combination therapy.

Ethics approval was granted by the Oxford Tropical Research and London School of Hygiene and Tropical Medicine ethics committees and relevant local ethical committees. Written informed consent was obtained from all patients or an attending relative.

### Measurements

Parasitemia was calculated for children with uncomplicated malaria, using the thick film parasite count per 200 leukocytes and the actual WBC count; if the WBC count was missing, the parasite count was calculated by assuming a WBC count of 8000 leukocytes/μL (count/200 leukocytes × 40) [10]. In the AQUAMAT study of severely ill children and the adult studies in Bangladesh and India, parasitemia was calculated from the thin blood film by examining 1000 red blood cells (count/1000 erythrocytes × 125.6 × hematocrit).

Baseline plasma specimens were collected in tubes containing ethylenediaminetetraacetic acid and stored at −80°C until testing. The method for measuring plasma *P. falciparum* HRP2 concentrations and resulting levels in the pediatric study have been described previously [3, 10].

### Plasma *P. falciparum* DNA Quantitation

To measure the parasite DNA concentration in each plasma sample, absolute quantitative real-time polymerase chain reaction (qPCR) analysis was performed directly by using plasma (2 μL) as a template without any DNA extraction step, 18S ribosomal RNA–targeting primers, and hydrolysis probes [13]. A Corbett Rotor-Gene-6000 cycler (Corbett Life Science, Sydney, Australia) was used with Quanti-Tect Multiplex PCR NoROX (Qiagen, Hilden, Germany) reaction mix. The PCR reaction mixture was prepared by using Quanti-Tect Buffer, 0.4 µM of each primer, and 0.2 µM of hydrolysis probe. The reaction conditions involved initial denaturation for 15 minutes at 95°C, 50 cycles of denaturation for 15 seconds at 94°C, and annealing for 60 seconds at 60°C.

A calibration standard was prepared using a highly synchronized culture of the 3D7 *P. falciparum* line. Suspensions containing precisely 10 000 ring-stage-infected red blood cells per tube were obtained using fluorescence-activated cell sorting, as described by Malleret et al [14]. The standard curves were linear (*r*^2^ > 0.98), with amplification efficiencies of 90%–105%. No nonspecific amplification products were seen on gel electrophoresis and by sequencing qPCR products from a range of field samples (Macrogen, Seoul, Korea). To check cross-contamination of samples during qPCR processing, negative controls (water) were added randomly (8 negative controls per 48 samples). Precision was assessed by 5 independent measurements of 5 samples spanning the range of DNA concentrations encountered (1.7 up to 37 000 genome equivalents/µL), with coefficients of variation ranging from 0.67% to 1.98% (cycle threshold), equivalent to 13.8%–38.2% (parasite genomes/µL).

### Statistics

Statistical comparisons between groups were performed using the Mann–Whitney test. Area under the receiver operating characteristic curve (AUROC) analyses for parasitemia, plasma concentrations of *P. falciparum* HRP2, and plasma concentrations of *P. falciparum* DNA were evaluated in Stata v 12.0 (Statacorp, USA), using logistic regression to calculate AUROC values for pairs of explanatory variables.

## RESULTS

Samples were obtained from 435 patients, comprising 224 children in Tanzania and Mozambique (111 with uncomplicated malaria and with 113 severe malaria, of whom 17 died) and 211 adults in Bangladesh and India (100 with uncomplicated malaria and 111 with severe malaria, of whom 33 died). Baseline characteristics of the 2 groups are shown in Table 1.

**Table 1.** Baseline Characteristics of African Children and Asian Adults With *Plasmodium falciparum* Malaria, by Malaria Severity

Characteristic	Children		Adults	
	Uncomplicated (n = 111)	Severe (n = 113)	Uncomplicated (n = 100)	Severe (n = 111)
Age, y	3.0 (1.9–4.2)	3.1 (1.5–4.9)	25 (20–40)	30 (22–42)
Female sex	50 (45)	57 (50)	28 (28)	32 (29)
Temperature, °C^a^	38.5 ± 1.0	38.2 ± 0.9	37.8 ± 1.1	38.3 ± 1.2
Hemoglobin concentration, g/dL	9.8 (9.0–11.1)	6.5 (4.6–8.4)	10.5 (7.9–12.8)	9.7 (7.0–11.5)
Microscopy positive for *P. falciparum*	103 (96)^b^	105 (99)^c^	97 (99)^d^	111 (100)
Parasitemia, parasites/µL	44 080 (17 720–81 120)	63 868 (14 789–201 965)	14 821 (2606–67 039)	40 186 (11 681–223 128)
Plasma *P. falciparum* HRP2 concentration, ng/mL	204 (95–435)	1690 (665–4271)	311 (123–763)	1973 (734–4263)
Plasma *P. falciparum* DNA concentration, genomes/µL	2.2 (0.6–12.2)	98.7 (13.0–635.3)	8.2 (0.8–36.2)	78.9 (25.7–1083)
Died	0 (0)	17 (15)	0 (0)	33 (30)

Data are no. (%) of patients, mean ± SD, or median (interquartile range).

^a^ Axillary in children, tympanic membrane in adults.

^b^ Data missing for 4 children.

^c^ Data missing for 7 children.

^d^ Data missing for 2 adults.

**Figure 1.**
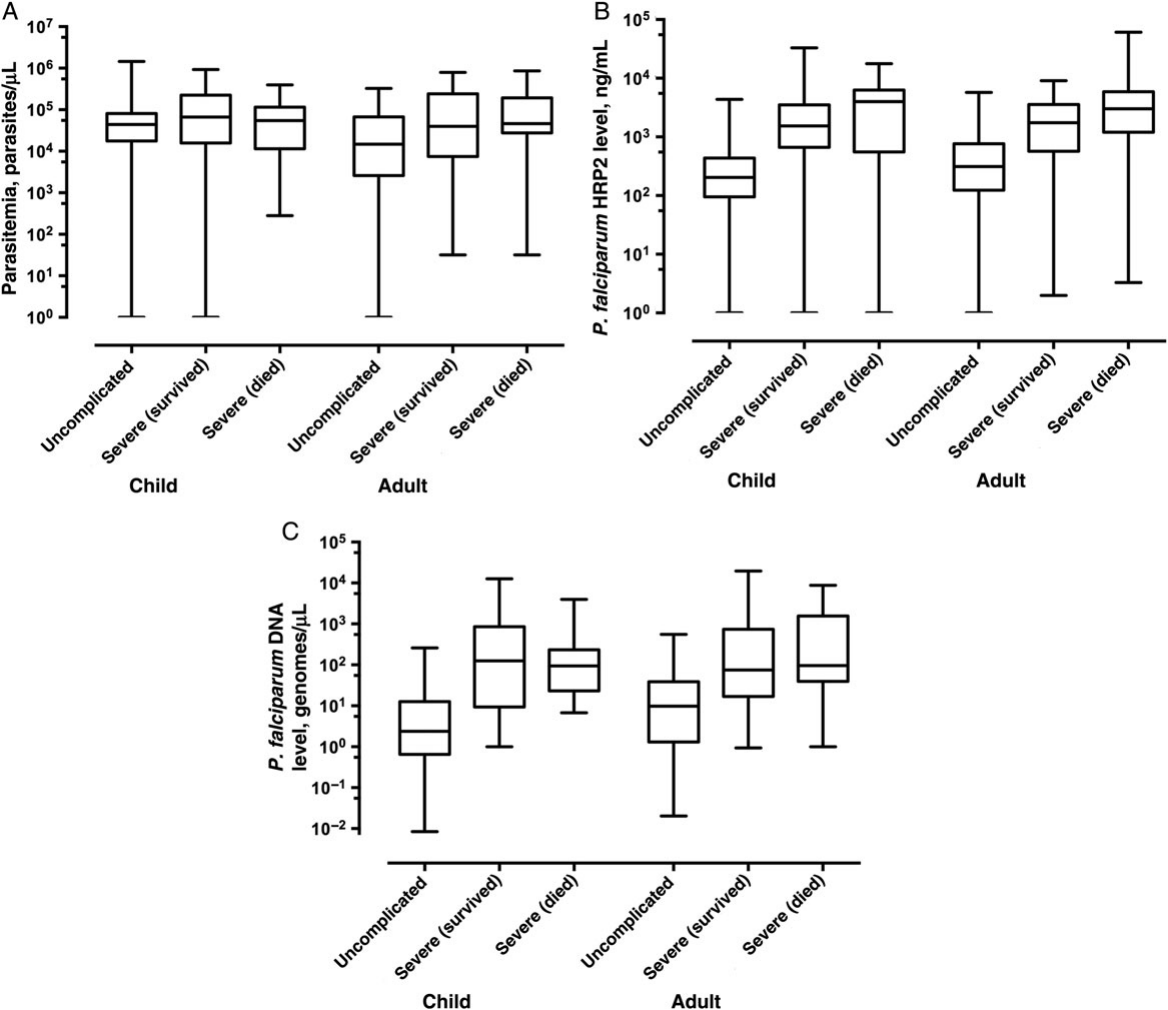
Parasitemia (*A*), plasma *Plasmodium falciparum* HRP2 concentration (*B*), and plasma *P. falciparum* DNA concentration (*C*) among African children and Asian adults who had uncomplicated malaria, who survived severe malaria, and who died from severe malaria.

Plasma *P. falciparum* DNA and *P. falciparum* HRP2 concentrations and peripheral parasitemia for the respective groups of patients are shown in Figure 1. Plasma *P. falciparum* DNA levels were approximately 40-fold and 10-fold higher in cases of severe malaria, compared with those in cases of uncomplicated malaria, in African children (median level, 98.7 genomes/µL in the severe group vs 2.22 genomes/µL in the uncomplicated group; *P* < .0001) and Asian adults (median level, 78.9 genomes/µL in the severe group vs 8.18genomes/µL in the uncomplicated group; *P* < .0001), respectively.

**Figure 2.**
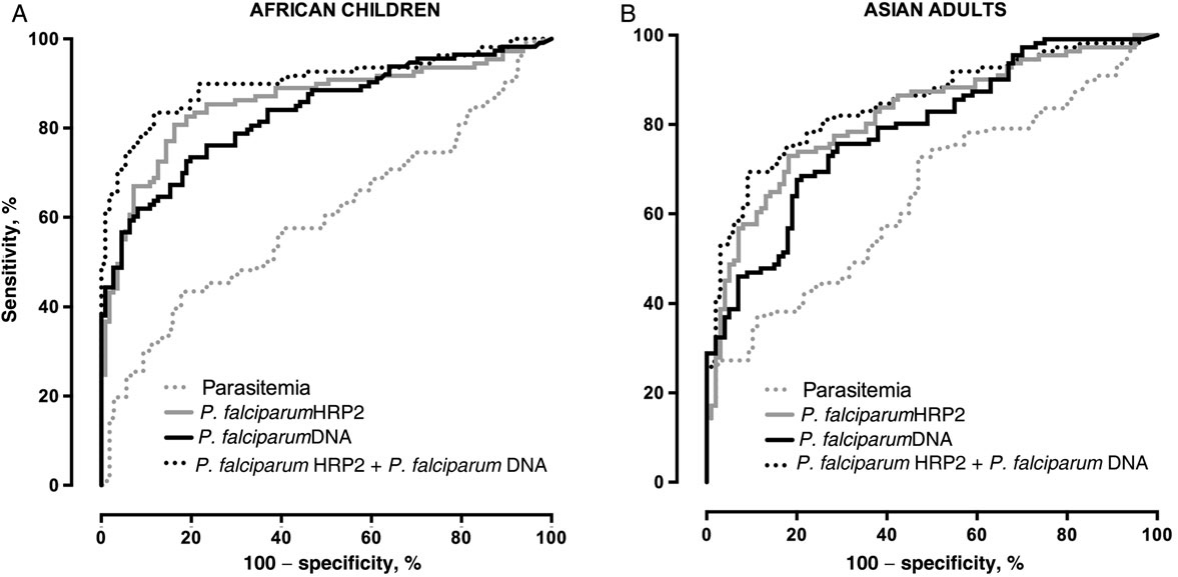
Receiver operating characteristic curves for test discrimination of uncomplicated from severe malaria in African children (*A*) and Asian adults (*B*) with *Plasmodium falciparum* malaria.

The AUROC values (Figure 2) for severe malaria were 0.834 and 0.789 for children and adults, respectively (Table 2). Analogous values for plasma *P. falciparum* HRP2 levels were 0.857 and 0.818, respectively. Both methods of assessment were substantially more accurate than parasitemia in differentiating severe from uncomplicated malaria (0.599 and 0.643, respectively; *P* < .0001 for both comparisons in both adults and children). There was no significant difference in AUROC values between plasma *P. falciparum* DNA concentrations and *P. falciparum* HRP2 concentrations (*P* = .43 and *P* = .29 for children and adults, respectively). Use of plasma *P. falciparum* DNA concentrations plus plasma *P. falciparum* HRP2 concentrations led to a further improvement in diagnostic accuracy, compared with use of *P. falciparum* HRP2 concentrations alone (for children, the AUROC increased to 0.904 [*P* = .004]; for adults, the AUROC increased to 0.847 [*P* = .003]). By comparison, use of parasitemia plus plasma *P. falciparum* HRP2 concentrations did not significantly improve the AUROC in either children (*P* = .5) or adults (*P* = .7).

**Table 2.** Area Under the Receiver Operating Characteristic Curve (AUROC) Values for Measures of Malaria Severity Among African Children and Asian Adults With *Plasmodium falciparum* Malaria

Measure	Children		Adults	
	AUROC	*P* Value^a^	AUROC	*P* Value^a^
Parasitemia	.599		.643	
*P. falciparum* HRP2 level	.857		.818	
*P. falciparum* DNA level	.834	.43	.789	.29
*P. falciparum* HRP2 and DNA levels	.904	.004	.847	.003
*P. falciparum* HRP2 level + parasitemia	.867	.51	.826	.68

^a^ Versus *P. falciparum* HRP2 level alone.

*P. falciparum* DNA concentrations in plasma specimens obtained at admission were not significantly associated with mortality within the severe malaria groups (for children, the median DNA level was 126 genomes/µL in survivors vs 94 genomes/μL in those who died [*P* = .96]; for adults, the median DNA level was 74.6 genomes/µL in survivors vs 96.3 in those who died [*P* = .42]). Parasitemia similarly did not predict outcome within the severe group in either location, whereas the plasma *P. falciparum* HRP2 level predicted outcome in Asian adults (median, 1741 ng/mL in survivors vs 3021 ng/mL in those who died; *P* = .009) but not in African children (median, 1535 ng/mL in survivors vs 3992 ng/mL in those who died [*P* = .148]).

## DISCUSSION

The presence of species-specific *Plasmodium* DNA in patients' plasma or serum has been recognized since the introduction of PCR methods for malaria diagnosis and genotyping [7, 8]. This nucleic acid derives presumably from degraded intraerythrocytic parasites and free merozoites. During the expansion phase of malaria, multiplication can exceed 50% efficiency (calculated as the parasite multiplication rate/average number of merozoites per schizont), but this rate falls abruptly at high densities, which must therefore generate a large number of free merozoites that are cleared by phagocytic cells. The relative contributions of merozoites and degraded intraerythrocytic parasites in producing the plasma *P. falciparum* DNA concentration are not known but could be tested by centrifugation or filtering to remove different sizes of particulate matter that contain parasite DNA. The kinetics of the production and elimination of plasma parasite DNA have not yet been characterized; studies of human DNA circulation in plasma indicate that rapid hepatic clearance occurs [15]. The half-life of fetal DNA in plasma is approximately 15 minutes [16].

Plasma concentrations of *P. falciparum* DNA reflected disease severity in falciparum malaria. Plasma *P. falciparum* DNA levels performed considerably better in both Asian adults and African children than peripheral parasitemia as a measure of disease severity and performed similarly to the recently validated plasma *P. falciparum* HRP2 as a severity measure [10]. The well-recognized poor performance of the peripheral blood parasite count as a severity measure is because the circulating parasites in falciparum malaria are not those causing the pathological processes; rather, the more mature sequestered parasites obstruct the microvasculature of vital organs. The number of these hidden pathological parasites is better reflected in the extraerythrocytic parasite products that circulate in plasma.

Importantly, the combination of plasma *P. falciparum* HRP2 and *P. falciparum* DNA concentrations was significantly more accurate than either measure alone. Additivity in accuracy of diagnostic performance suggests that the 2 markers provide independent measures of severity. This may reflect differing intrinsic mechanisms and kinetics of production and elimination. It might also result from covariates affecting either marker that are independent of parasite biomass, so that the 2 measures complement each other. Potential covariates include *P. falciparum* HRP2 gene deletion [17] (although our previous survey based on plasma *P. falciparum* HRP2 levels in African children found no evidence of this [18]), differential *P. falciparum* HRP2 expression [19], and small copy number variations in the rDNA units containing the 18S (SSU) sequences used to detect parasite DNA [20, 21]. Despite its strong association with severity, the plasma parasite DNA level did not predict mortality in either group of patients. The reasons are unclear; possible explanations include a temporal dissociation between microvascular obstruction and DNA release or a stronger association between high DNA concentrations and less severe syndromes of severe malaria.

A clinical definition of severe malaria was used as the gold standard for calculations of diagnostic accuracy (measured as the AUROC). However, in the AQUAMAT study (in which the severe pediatric cases were studied), one third of cases (defined by the ranking of plasma *P. falciparum* HRP2 concentrations) had no mortality benefit from artesunate, compared with quinine, which suggests that they may have had another lethal disease (most likely invasive bacterial infections) [3]. This suggests that the gold standard for comparative evaluation is imperfect and that, in children with clinically diagnosed severe malaria, there is a theoretical maximum diagnostic accuracy, as specificity is diluted by cases in which malaria is not actually the cause of severe illness. Accuracy in clinical trials would improve with stricter definitions involving confirmation of malarial retinopathy, but this applies only to cerebral malaria, which usually composes less than half of the cases presenting with severe malaria, and so would lack sensitivity [22]. In low-transmission settings where adults develop severe malaria, coincidental peripheral blood parasitemia is less frequent; therefore, among ill adults, parasitemia has good specificity for malaria, although parasite density is still relatively inaccurate as a severity measure. The smaller differences in plasma *P. falciparum* DNA concentrations between the uncomplicated and severe cases among adults, compared with those observed among children, in this study may be explained by disease severity. The adults with uncomplicated malaria studied in Bangladesh and India were sufficiently ill to warrant admission to a referral hospital (ie, a small minority of cases overall), had higher plasma *P. falciparum* HRP2 and *P. falciparum* DNA concentrations, and probably had more-severe infections than the African children with uncomplicated malaria recruited in the community-based studies.

Quantitation of plasma *P. falciparum* DNA concentrations is currently unlikely to be incorporated into prospective patient management, although rapid advances in PCR technology may change this. The *P. falciparum* DNA concentration is a useful prognostic measure in research studies and may provide valuable information from previous studies in which plasma or serum samples have been archived. qPCR can be readily adapted to a high-throughput format. Plasma measurements reflecting the sequestered parasite burden are valuable indicators of disease severity in falciparum malaria.
